# RNA-seq of HaHV-1-infected abalones reveals a common transcriptional signature of *Malacoherpesviruses*

**DOI:** 10.1038/s41598-018-36433-w

**Published:** 2019-01-30

**Authors:** Chang-Ming Bai, Umberto Rosani, Ya-Nan Li, Shu-Min Zhang, Lu-Sheng Xin, Chong-Ming Wang

**Affiliations:** 1 Key Laboratory of Maricultural Organism Disease Control, Ministry of Agriculture; Laboratory for Marine Fisheries Science and Food Production Processes, Qingdao National Laboratory for Marine Science and Technology; Qingdao Key Laboratory of Mariculture Epidemiology and Biosecurity; Yellow Sea Fisheries Research Institute, Chinese Academy of Fishery Sciences, Qingdao, 266071 China; 2 Department of Biology, University of Padua, Padua, 35121 Italy; 3 College of Fisheries, Tianjin Agriculture University, Tianjin, 300380 China; 4 College of Fisheries and Life Science, Dalian Ocean University, Dalian, 116023 China

## Abstract

*Haliotid herpesvirus-1* (HaHV-1) is the viral agent causative of abalone viral ganglioneuritis, a disease that has severely affected gastropod aquaculture. Although limited, the sequence similarity between HaHV-1 and *Ostreid herpesvirus-1* supported the assignment of both viruses to *Malacoherpesviridae*, a *Herpesvirales* family distantly related with other viruses. In this study, we reported the first transcriptional data of HaHV-1, obtained from an experimental infection of *Haliotis diversicolor supertexta*. We also sequenced the genome draft of the Chinese HaHV-1 variant isolated in 2003 (HaHV-1-CN2003) by PacBio technology. Analysis of 13 million reads obtained from 3 RNA samples at 60 hours post injection (hpi) allowed the prediction of 51 new ORFs for a total of 117 viral genes and the identification of 207 variations from the reference genome, consisting in 135 Single Nucleotide Polymorphisms (SNPs) and 72 Insertions or Deletions (InDels). The pairing of genomic and transcriptomic data supported the identification of 60 additional SNPs, representing viral transcriptional variability and preferentially grouped in hotspots. The expression analysis of HaHV-1 ORFs revealed one putative secreted protein, two putative capsid proteins and a possible viral capsid protease as the most expressed genes and demonstrated highly synchronized viral expression patterns of the 3 infected animals at 60 hpi. Quantitative reverse transcription data of 37 viral genes supported the burst of viral transcription at 30 and 60 hpi during the 72 hours of the infection experiment, and allowed the distinction between early and late viral genes.

## Introduction

The viral family of *Malacoherpesviridae* is a divergent group of the Herpesvirales order^1^, consisting in two ICTV (International Committee on Taxonomy of Viruses)-accepted members, *Ostreid herpesvirus 1* (OsHV-1^2^) and *Haliotid herpesvirus 1* (HaHV-1^3^). Crassostrea gigas, a bivalve, and *Haliotis* spp., gastropods, are the prevalent hosts of OsHV-1 and HaHV-1, respectively. However, these viruses display a broad host-range and their presence was reported in a number of mollusk species^4–6^. Up to now, *Malacoherpesviridae* are the only known herpesviruses infecting invertebrates, although the presence of herpesvirus-like particles associated to the king crab was recently reported^7^. Undoubtedly, *Malacoherpesviridae* possess some typical features of Herpesvirales (reviewed in^8^), but, since a limited number of Herpesvirales-ortholog genes have been identified in their genomes, the evolutionary history of *Malacoherpesviridae* is still under debate. Recent analyses suggested that *Malacoherpesviridae* capsid proteins and enzymes are related to genes found in bacterial and archaea dsDNA viruses^8,9^ and, the identification of large OsHV-1 genome regions in the assembled genomes of *Brachiostoma* spp. (Chordata) and *Capitella teleta* (Annelida) has added complexity to the evolutionary trajectories of these viruses^3,10^.

The first identification of an OsHV-1-like viral particle was reported in 1972, associated to oyster mortalities^11^, whereas HaHV-1 was firstly reported in 2003 in Taiwan and subsequently in 2005 in Australia. In both cases, HaHV-1 was associated to massive mortalities described as abalone viral ganglioneuritis (AVG) due to typical neuropathological signs^12,13^. HaHV-1 was also suspected as the etiological agent of an epizootic disease that wiped out the entire abalone farming industry in southeastern China during 1999 and early 2000s^14^. High amounts of HaHV-1 DNA have been detected in samples of diseased *H. diversicolor supertexta* collected between 1999 and 2003 in China^15^. It has also been inferred that HaHV-1 originated from the Chinese mainland and was then transferred to Taiwan^16^ from where it was transported to Australia through Taiwanese Abalone Feed (Dr. J. Thyer, unpublished data). Overall, the emergence of more aggressive malacoherpesvirus variants has raised great concerns in the mollusk’s aquaculture sector^17–21^ and, in this context, the use of ‘omics approaches have the potential to disentangle complex molecular interactions between the host and the pathogen and could help the understanding of the disease onset^22^.

The analysis of RNA obtained from OsHV-1 infected animals by suppression subtractive hybridization (SSH)^23^ and high-throughput (HT) sequencing^24,25^ provided the first transcription data on the OsHV-1 genes. Thanks to the latter approach, an almost complete landscape of the OsHV-1 transcriptome was produced and, at the same time, the viral reads were employed to finely characterize OsHV-1 variants in the absence of a proper reference genome^10^. Later, HT-DNA sequencing was used to sequence a number of OsHV-1 micro-variants from different European locations^26,27^.

Interestingly, before the analysis of OsHV-1-infected samples, little was known about oyster antiviral immunity and analyses often founded on HT approaches have greatly improved the knowledge on mollusk’s antiviral pathways^24,25,28–31^. The antiviral elements in *C. gigas* have been greatly revealed and facilitating comparative characterization among other bivalve species^32^. Likewise, the study of gastropod antiviral defenses will greatly benefit from the availability of genetic information on HaHV-1 infections. Moreover, the concurrent investigation of OsHV-1 and HaHV-1 viruses represents a great opportunity to understand host-pathogen co-evolutionary processes in phylogenetically distant marine invertebrates.

In the present study we firstly employed an RNA-seq approach on HaHV-1-infected abalones to report transcriptional data of HaHV-1 at 60 hpi. Coupling of RNA and DNA HT-sequencing permitted us to substantially improve the HaHV-1 genome annotation, according to the annotation of new viral genes and the identification of several nucleotidic variations. Secondly, quantitative reverse transcription PCR (qRT-PCR) was used to investigate the expression pattern of 37 viral genes during the 72 hours of the infection experiment.

## Results

**Figure 1:**
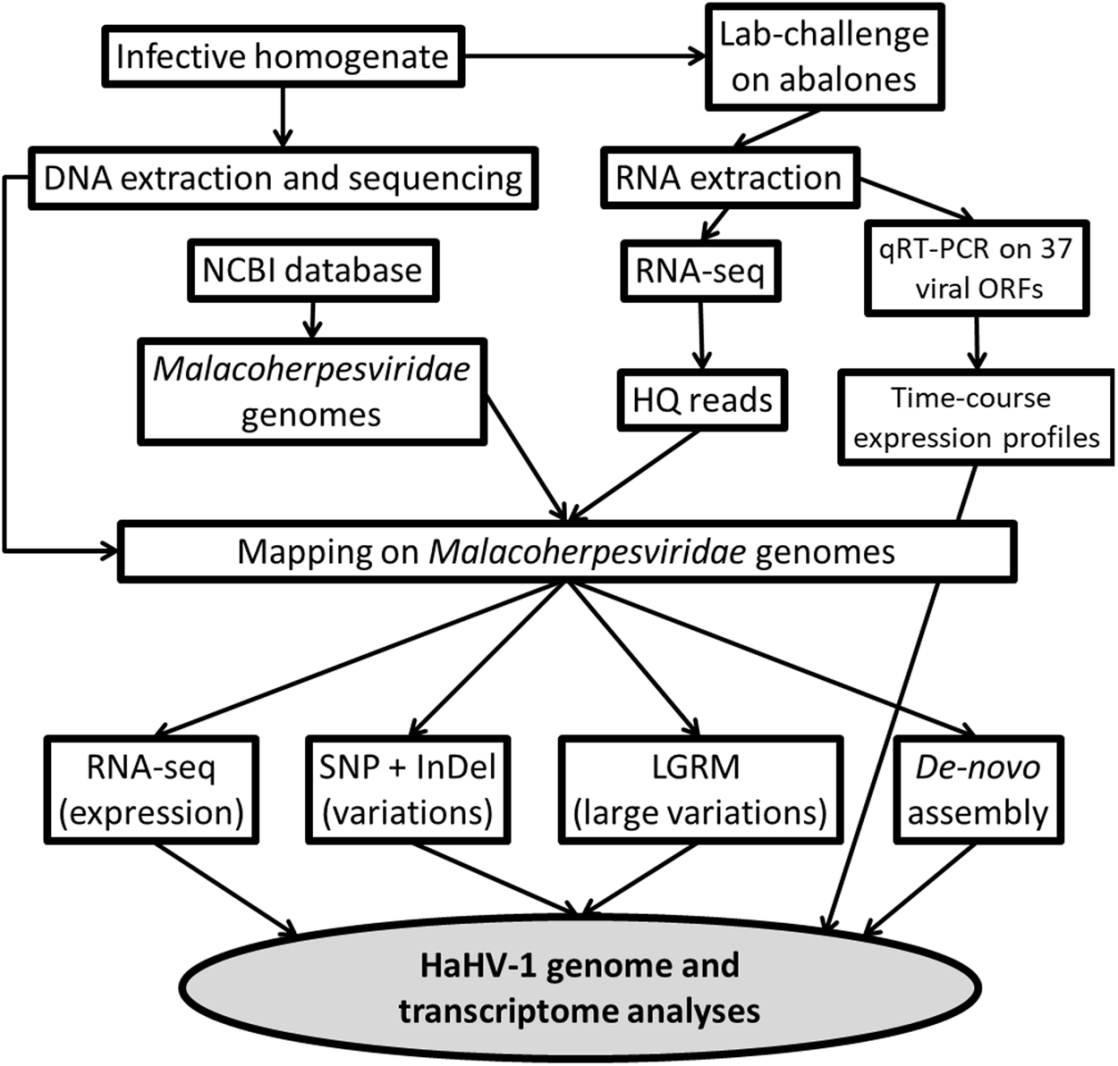
The experimental and analytical pipeline for this work.

The mortality curve and the increasing viral DNA amounts in all the 4 analyzed tissues demonstrated the infection effectiveness of the HaHV-1 injection in this batch of *H. diversicolor supertexta*, whereas no mortality was observed in the control group (Supplementary Fig. 1). According to the increased amount of viral DNA as well as to the observation of the first dead abalones, we selected 3 abalones from the 60 hpi time-point for high-throughput transcriptomic analysis. RNA sequencing produced a total of 145.3 M of high quality (HQ) paired reads (Table 1), that were deposited at the NCBI SRA Database (accession ID: PRJNA471241). By mapping the HQ reads on the 8 available *Malacoherpesviridae* genomes, we identified 12.8 M reads of viral origin (Table 1). All these viral reads pertained to 2 gastropod *Malacoherpesviruses*, since no one read mapped on the genomes of bivalve *Malacoherpesviruses*. Moreover, most of the viral reads (74–75%, depending on the sample), mapped to the genome of the HaHV-1-Taiwan variant, whereas the remaining reads showed a higher similarity to the HaHV-1-AUS genome (Supplementary Table 1). The experimental and analytical pipeline adopted in this study is depicted in Fig. 1.

**Table 1:** Sequencing results.

Sample ID	Description	No. of HQ reads [M]	No. of viral reads
MA_49	60 hpi	50.0	4,121,744
MA_50	60 hpi	40.5	3,416,288
MA_51	60 hpi	54.8	5,523,471

Sample ID and description, number of high quality reads in millions and number of viral reads per sample were reported.

### HaHV-1 genome analysis

Prior to other analyses, we exploited the viral reads to improve the annotation of the viral genome (GenBank ID: KU096999.1, hereinafter abbreviated to HaHV-TAI). Depending on the applied mapping algorithm and mapping parameters (see M&M for additional details), a range of 12.7–13.1 M reads per sample mapped on the reference genome (Supplementary Table 2). To reconstruct the transcriptionally active genome regions, we *de-novo* assembled the viral reads, obtaining 25 contigs with a N50 equal to 13,964 nt and with a length range spanning from 524 to 34,404 nt (accession ID: PRJNA492770). Using the same constraints applied for ORF prediction in other *Malacoherpesviridae* genomes^2,26,27^, we could identify 112 ORFs in these 25 contigs (called ‘*de-novo*-derived’ ORFs).

Applying the same parameters, we screened the viral genome for the presence of not annotated ORFs. As a result, we predicted 51 new ORFs, which increased the number of HaHV-TAI ORFs to 117, and we estimated around 80% of the HaHV-TAI genome length to be transcribed (hereinafter, this newly annotated genome is called ‘HaHV-TAI_impr’). This number (117) is similar to that of the annotated ORFs of other *Malacoherpesviridae*, and it rectifies the lower ORF number previously annotated on the HaHV-TAI genome (refer to Fig. 1 in^10^). The HaHV-TAI_impr ORFs encoded a total of 27 conserved protein domains (for a total of 21 different domains). Comparing them with the conserved domains predicted in the *de-novo*-derived ORFs and with the ones present in the HaHV-AUS predicted proteins, we demonstrated that both *de-novo* and genome re-annotation approaches allowed the recovery of almost all the recognizable viral protein domains (Supplementary Fig. 2). Furthermore, we observed that the dUTPase and eIF-5_eIF-2 domains were not present in the *de-novo*-derived ORFs and 3 other domains (OrfB_Zn_ribbon, OrfB_IS605 and HTH_OrfB_IS605, co-occurring on HaHV-AUS ORF86) were not present in the HaHV-TAI genome. Interestingly, HaHV-AUS ORF86 was suggested to originate from a recent horizontal transfer from bacteria^8,10^. Besides, 4 of the newly predicted ORFs contain a signal peptide region (increasing to 11 possible secreted proteins of HaHV-TAI). Supplementary Data 1 includes the HaHV-TAI_impr genome annotation.

### Viral genome sequencing

The DNA extracted from the viral inoculum used to perform the abalone laboratory challenge was subjected to high-throughput DNA sequencing based on PacBio-sequencing of genomic amplicons generated by 21 Long-Range PCRs. As a result, we obtained 79,940 HQ reads (mean length 15,183 nt), which were assembled in 5 contigs (N50 = 59,009), representing the first genome draft of the HaHV-1 2003 variant (Guangdong Province), called HaHV-1-CN2003. The draft genome of HaHV-1-CN2003 is available in Supplementary Data 2.

### SNP and InDel analysis

**Figure 2:**
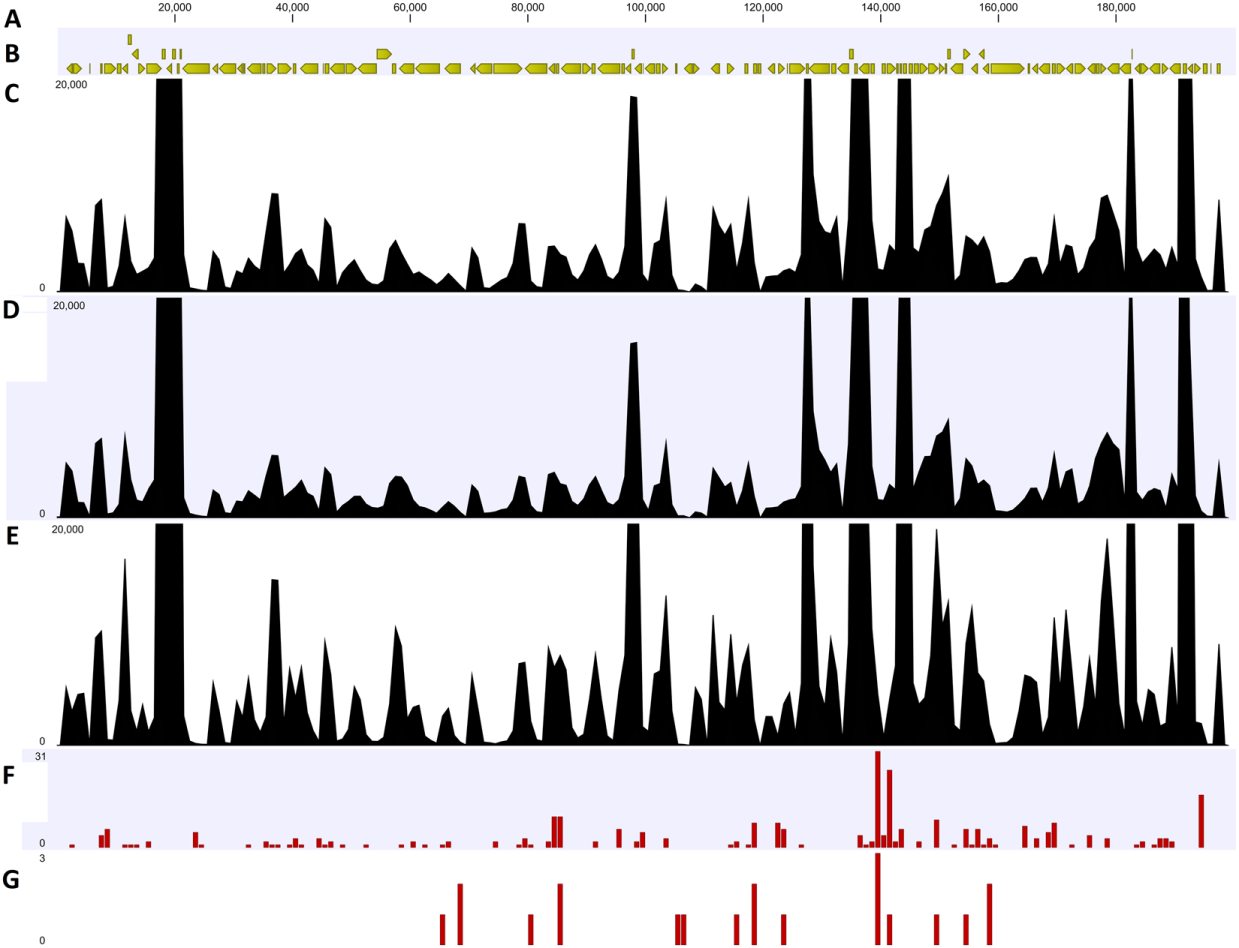
(**A**) HaHV-1_impr viral genome; (**B**) predicted ORFs; (**C**–**E**) coverage graph for sample MA49, MA50 and MA51; (**F**) distribution of common SNPs and (**G**) distribution of common InDels along the genome.

Exploiting the large number of HaHV-1 viral RNA reads and the genome draft, we proceeded to identify sequence variations, i.e. SNPs and Insertions or Deletions (InDels) at both transcriptomic and genomic levels. SNP calling using the HaHV-TAI as references identified 227, 216 and 253 variable positions in MA49, MA50 and MA51, respectively, for a total of 192 common variable positions. InDel analysis identified 20 common variations, consisting in 13 deletions (23–163 bp) and 7 insertions (6–27 bp), with a total of 7 InDels located within coding regions (Table 2, Supplementary Table 3). Sixty seven percent of the total variable positions were common between the 3 datasets, although this percentage increased if we considered only the coding SNPs (95% of them are common) or the non-synonymous SNPs (nsSNPs, 84%). Actually, 64% of the 192 common SNPs were located outside coding regions, while, 79% of the 103 common coding SNPs were nsSNPs. Joining SNP and InDel analyses, we identified 207 variable positions: 135 SNVs (single nucleotide variations, consisting in SNPs or 1 nucleotide InDels), 41 larger insertions and 31 deletions (Fig. 2).

**Table 2:** Variation analysis.

Sample	No. of SNPs	No. of coding SNPs	No. of nsSNPs	No. of InDels
MA_49	227	107	71	41
MA_50	216	109	74	41
MA_51	253	113	76	62

Number of total SNPs, coding SNPs, nsSNPs and InDels for each sample were reported.

Exploiting the genome draft generated by PacBio sequencing, we traced the variable positions identified by the RNA data on the viral genome. A total of 113 variations were confirmed by genome data, for 60 variations the genome consensus was invariant (i.e. equal to HaHV-TAI genome) and 33 variations were not covered by PacBio data. Interestingly, 42% of the transcriptomic variations (e.g. the ones not supported by genome data) represented T to C transitions and were located in a genomic hotspot (HaHV-TAI: 194,743:194,903, Supplementary Table 3).

Mapping the viral RNA reads on the HaHV-1-CN2003 genome draft (the consensus obtained from the same samples and likely representing the same virus), we could identify the transcriptomic variations, consisting in 89 SNPs mostly located outside coding regions (87%). Intriguingly, only 36 variations were commonly found in the 3 RNA datasets, whereas 43 variations were present only in one dataset and 10 were found in two datasets.

### Phylogenetic analysis

**Figure 3:**
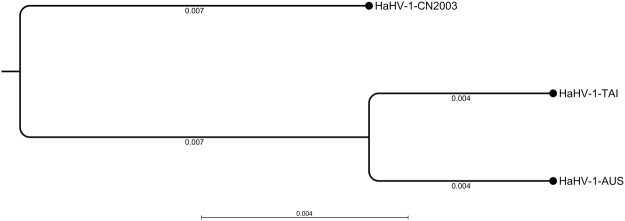
NJ phylogenetic tree based on 40 concatenated ORFs retrieved from the 3 gastropod *Malacoherpesviridae* genomes. Phylogenetic distances were reported under the corresponding branches.

We concatenated 40 homologous ORFs retrieved from the 3 gastropod *Malacoherpesvirus* genomes to run a phylogenetic analysis based on the Neighbor joining (NJ) algorithm. Although the result is limited by the small number of available viral variants, the phylogenetic tree based on 57,728 aligned positions may suggest that HaHV-1-CN2003 represents the ancestral viral variant from which the other 2 known variants arose (Fig. 3).

### Expression of viral genes

**Figure 4:**
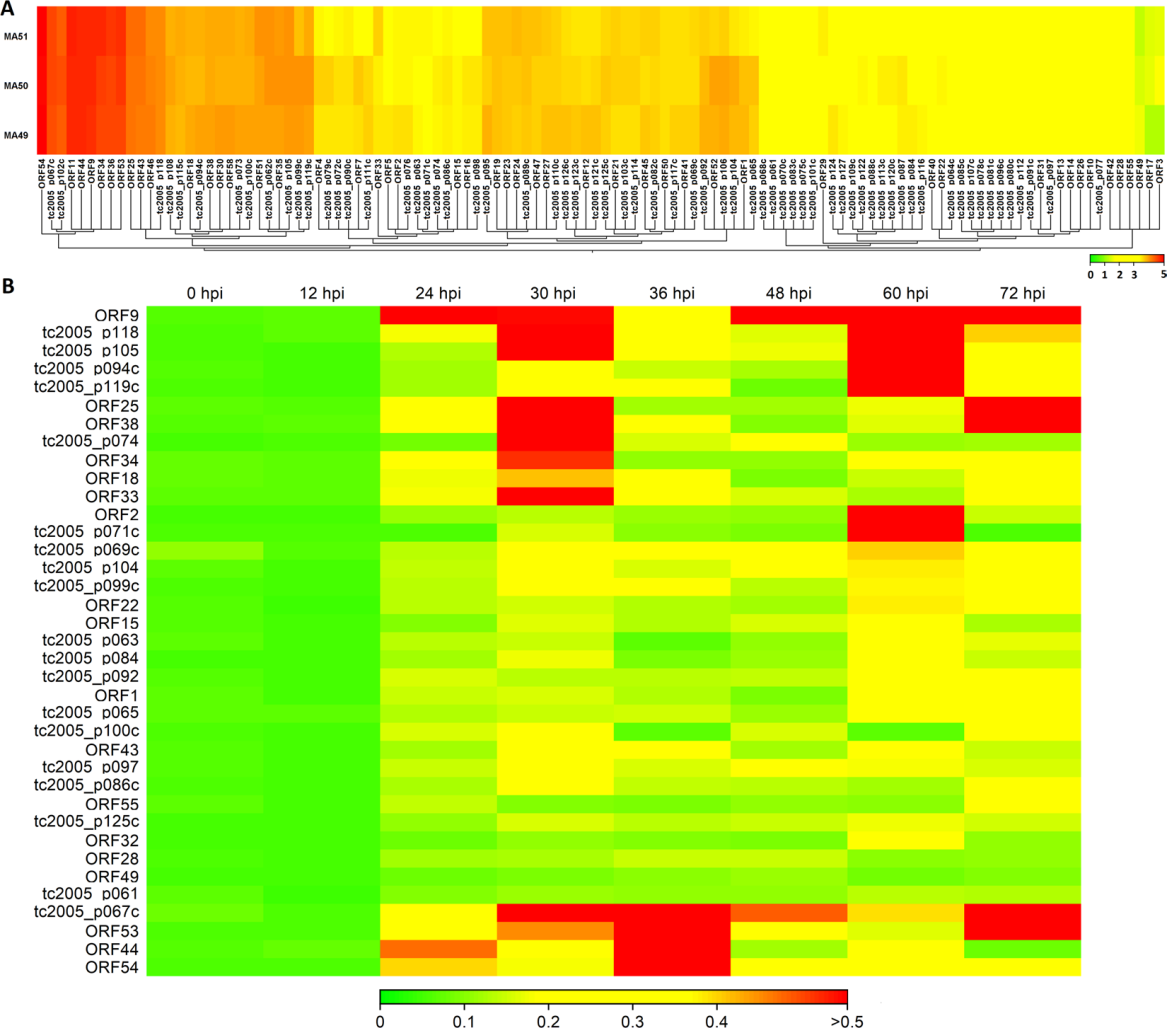
(**A**) Heat map representing viral TPM expression values over the 3 RNA samples at 60 hours post injection (hpi). (**B**) qRT-PCR expression data of 37 viral genes. The averaged expression values of 3 biological replicates per time point were reported as 1/delta Ct.

We further exploited the viral RNA reads to study the viral expression profiles in the 3 infected abalones sampled at 60 hpi. We used the HaHV-TAI_impr genome as a reference for RNA-seq analysis, to calculate the expression values for each of the 117 predicted viral genes. The expression profiles were similar for the 3 samples (Fig. 4A and Supplementary Table 4) and most of the 117 predicted ORFs were supported by measurable expression values in all the 3 RNA samples, while 8 ORFs (7 newly predicted ones) showed a negligible number of mapped reads (below 100). Although the support of expression data is an indication of the existence of a viral gene^33^, we decided to consider these ORFs as ‘putative’, since the transcription boundaries (the real start and stop positions of each transcript) remained undefined. Figure 2 (graph C, D and E) demonstrated the consistent presence of several expression peaks among the 3 expression profiles. Analyzing in deep these coverage graphs, it was evident that most of the ORFs are transcribed in separate transcriptional units, whereas we reported the possible presence of a polycistronic mRNA including 4 predicted ORFs (p103-p104-p105-p106, Supplementary Fig. 3A). This coverage graph greatly differs from that of ORF53, taken as an example of a well-defined viral mRNA (Supplementary Fig. 3B). Among the 20 most expressed ORFs, we found a small secreted protein (ORF54, 240 aa), a putative envelope protein (p119), 2 putative capsid proteins (p067c and p099c) and the putative capsid maturation protease called assemblin (p102, Table 3). Moreover, a gene containing a HUH endonuclease fused with a helicase was also present (p105), whereas the other highly expressed ORFs were genes with an unknown function. Among these 20 genes we could trace 9 OsHV-1 orthologues, all of them showing high expression levels in virally-infected oysters^10^.

**Table 3:** Expression values.

ORF ID	Annotation	Similarity to		Notes	Expression values (TPM)			
		HaHV-AUS	OsHV-1		MA49	MA50	MA51	Average
ORF54	\	ORF110	\	secreted	129584,8	99790,4	119907,2	116427,5
ORF11	\	ORF16	ORF80	\	62247,0	68937,8	73577,2	68254,0
ORF9	\	ORF13	\	\	59218,3	66402,4	67147,6	64256,1
ORF44	\	ORF78	\	\	61965,2	64836,9	65049,4	63950,5
ORF34	\	ORF67	ORF83	\	42145,9	44092,2	69043,4	51760,5
ORF53	\	ORF104	\	\	43110,7	49459,7	56710,9	49760,4
ORF36	\	ORF72	\	\	40452,2	40973,7	54989,6	45471,8
tc2005_p067c	capsid protein	ORF14	ORF82	\	37353,7	41959,6	41413,5	40242,3
tc2005_p102c	assemblin	ORF73	ORF107	\	28754,3	34386,8	30319,7	31153,6
ORF25	\	ORF51	\	\	19773,4	20824,4	23168,7	21255,5
ORF43	\	ORF77	\	\	17557,3	20020,1	21325,3	19634,2
ORF46	\	ORF84	\	\	16761,0	16671,5	13479,3	15637,3
tc2005_p118	\	ORF101	ORF24	\	13976,9	13719,3	14424,0	14040,0
tc2005_p105	HUH endonucl.	ORF82	\	\	10958,0	12180,4	11828,6	11655,7
tc2005_p062	\	ORF7	ORF91	\	9463,2	12380,7	12360,0	11401,3
ORF35	\	ORF71	ORF89	\	10377,9	11801,9	10899,6	11026,5
tc2005_p119	envelop fusion protein	ORF102	ORF68	\	10075,0	12126,7	8875,1	10358,9
ORF51	\	ORF95	\	\	9369,7	9578,0	11928,2	10292,0
tc2005_p099c	capsid protein	ORF68	ORF104	\	10657,8	11853,6	6655,2	9722,2
ORF38	\	ORF75	\	\	10423,0	9042,1	9058,7	9507,9

ORF ID, putative annotation, similarity to HaHV-1 and OsHV-1 ORFs, additional features and TPM expression values in the 3 samples as well as averaged expression for the top 20 expressed ORFs were reported.

To better contextualize the 3 expression profiles obtained at 60 hpi by RNA-seq, we traced the expression of 37 selected viral genes at 8 time points post injection (Supplementary Table 5). The heat-map depicted the averaged qRT-PCR expression data per time point (Fig. 4). The HaHV-1 transcription massively started at 24 hpi, since almost all tested viral genes showed similar expression values to those of the control ones at 12 hpi (Fig. 4B). The cumulative expression of the 37 genes is 2.3, 2.1, 6.3, 12.6, 9.2, 6.0, 12.8 and 10 at 0, 12, 24, 30, 36, 48, 60 and 72 hpi (Supplementary Table 5), respectively, supporting the existence of 2 main viral expression time points that is at 30 and 60 hpi (Fig. 4B). We observed that the viral genes showed an ‘early’ expression peak (e.g. peaking their expression at 24, 30 or 36 hpi) always followed by a second peak at 60 hpi or 72 hpi. Some viral genes showed a ‘late’ peak at 60 or 72 hpi, and some genes appeared over-expressed along all the infection period. Notably, 2 putative envelop proteins showed alternative expression, with p074 peaking its expression at 30  hpi and p119c at 60 hpi. Three capsid proteins (p067c, p099c and p104) showed a consistent expression trend at 30 and 60 hpi, with p067c was the highest expressed ones. The secreted protein ORF54 as well as the other ORFs with unknown functions expressed at the early stage of HaHV-1 infection.

## Discussion

Irrespective of their unsolved origin and partially uncharacterized phylogenetic relationships with other viruses, *Malacoherpesviruses* represent an authentic concern for aquaculture^17,26,34–38^. In particular, HaHV-1 greatly challenged the abalone production in China as well as in other regions^4,12^. Similar to the bivalve OsHV-1^36,37^, HaHV-1 infects multiple hosts showing susceptibility to the virus^4,12,39^.

In this paper, for the first time, we reported HaHV-1 transcriptional data obtained from messenger RNA sequencing of 3 experimentally infected *H. diversicolor supertexta* sampled at 60 hpi, and we corroborated these transcriptomic data with qRT-PCR expression data obtained from 8 time points during the 72 hours of the experimental infection. Notably, the percentage of viral reads recovered from the 3 HaHV-1-infected samples (8–10% of total ones) was much higher than that recovered from OsHV-1-infected *C. gigas* (Supplementary Fig. 4)^40^. It becomes evident that we detected a surprising amount of viral reads, which suggested a sharp accumulation of viral transcription at 60 hpi. qRT-PCR data supported the sustained viral expression both at 30 and 60 hpi, while limited viral expression at 24, 36 and 48 hpi.

Up to now, only 2 gastropod *Malacoherpesvirus* genomes have been characterized, although it is likely that additional viral variants are present. We exploited the high number of viral RNA reads, coupled with a viral genome draft obtained using PacBio sequencing, to characterize the HaHV-1-CN2003 viral variant isolated in the Guangdong Province (China). Firstly, we demonstrated that the Taiwanese HaHV-1 variant is the most similar variant and, accordingly, we improved its genome annotation with a total of 117 ORFs covering most of the viral genome. Arguably, the absence in HaHV-1-CN2003 and HaHV-1-TAI of the ORF86 found in HaHV-1-AUS and probably originated from a bacterial transfer^8^, may suggest that the latter virus derive from one of the other two. Phylogenetic analysis of these 3 viral genomes can only state the higher distance of HaHV-1-CN2003 compared to the distance between the Taiwanese and the Australian viral variants. To ascertain the evolutionary history of these viruses there is the need of data regarding a higher number of viral variants.

We further characterized the HaHV-1-CN2003 genome with 207 variable positions, which differentiated this genome from the HaHV-1-TAI genome. Although all these variants are common to all the 3 RNA datasets and likely represented genomic variations, we could validate only a part of them (113) by using paired DNA data due to the incompleteness of the genome draft obtained in the present study. In addition, we demonstrated the presence of viral transcriptional variability by the direct comparison of RNA-seq and DNA data. The 60 transcriptomic variations could have arisen from the presence of multiple viral variants or, more likely, from low-fidelity viral-transcription^41^. However, since several RNA variable positions were commonly found in 3 different samples, it is possible that the transcriptional variability is generated around hotspots. In fact, most of the DNA-unverified SNPs resulted to be T to C transitions located in hotspots. Notably, most of the coding SNPs are located in few ORFs and could be classified as nsSNPs, symptomatic of a selective pressure acting on certain viral genes. At present, it is not clear if multiple viral variants co-occurred in the same infected animal or within the same batch of infected animals. Contrasting data, sometimes biased by the absence of the proper reference genome, either suggested or excluded the presence of co-occurring viral variants^10,26^. Using SNP and InDel analysis based on paired RNA and DNA data, we cannot definitively exclude the presence of more than one viral variant within the 3 analyzed abalones.

RNA data mining supported the translation into viral proteins of almost all ORFs, although proteomics approaches are needed to definitively resolve the annotation of the viral proteins. The expression analysis of the viral genes revealed highly overlapping patterns in the 3 infected abalones at 60 hpi. Such patterns are also somewhat conserved between HaHV-1 and OsHV-1, and this quite surprising result suggests that the roles played by the most expressed ORFs of both viruses are evolutionary conserved. In support of this hypothesis, the most expressed viral genes were putatively involved in the capsid formation and maturation. Since most of the *Malacoherpesviridae* ORFs do not found matches in the sequence databases, transcriptional data would support their active roles during viral infection, and thus contribute to the selection of viral targets for functional validation through the production of recombinant proteins. With this aim, we traced the expression of 37 viral genes in the mantle tissue over the whole time-course of an experimental infection. Surprisingly, although most of the viral genes appeared expressed after 24 hpi and along all the infection period, some of them are characterized by two expression peaks, at 30 hpi (early infection) and 60 or 72 hpi (late infection), and limited expression at 48 hpi. Putative capsid and envelop viral proteins consistently followed this 2-peaks expression trend. These time-course expression data partially agreed with those reported during OsHV-1 infection^24^. Differently from that work, most of the HaHV-1 genes peaked their expression after the 24 hpi point and we do not observe a decreased viral expression after 48 hpi. However, since we analyzed more points in a shorter time allotment, this could possibly be the reason of such difference.

In conclusion, we have described the commonalities of gene expression and sequence characteristics within *Malacoherpesviruses*, suggesting strict constraints between the expression level and function of most of the highly expressed ORFs. Expression results supported the burst of the viral DNA observed from the 24 hpi and the presence of two peaks of viral transcription and virion assembly. The integrated analysis of *Malacoherpesvirus* ORFs would contribute to the selection of *Malacoherpesvirus* gene targets for functional studies.

## Methods

### Animals and experimental infection

All the protocols of animal handling and sampling were approved by the Animal Care and Ethics Committee, Yellow Sea Fisheries Research Institute, Chinese Academy of Fishery Sciences. All the methods were carried out in accordance with the approved protocols and relevant guidelines.

Four hundred virus-free *H. diversicolor supertexta* (size range between 49.73 and 58.24 mm) were bought from Xiamen, Fujian Province, and transferred to Qingdao, Shandong Province of the China country by air in April 18^th^, 2016. These abalones were maintained in 50 L tanks (40 abalones per tank) supplied with aerated, filtered seawater and adequate seaweed (*Laminaria japonica*). At the end of the two-week of acclimation period, 30 animals were selected randomly and tested negative for HaHV-1 DNA. The salinity and temperature of water were fixed at 30 ± 1 ppt and 19 ± 1 °C, respectively and half-changed daily throughout the experiment.

A viral inoculum was prepared from *H. diversicolor supertexta* found infected with high HaHV-1 loads, originally collected from abalone farms in the Guangdong Province in 2003. The standard protocol for the OsHV-1 inoculum preparation, described in^42^, was employed to prepare tissue homogenates, except that 0.22 µm-filtered natural seawater instead of artificial seawater was used in all dilution steps. Tissue homogenates for negative controls were prepared using HaHV-1-negative *H. diversicolor supertexta* with the same protocol. Filtered tissue homogenates were stocked at 4 °C until use. An aliquot of each tissue homogenate (200 µL) was used for HaHV-1 DNA detection and quantification by quantitative PCR (qPCR) (described below).

For the experimental infection, abalones were firstly anaesthetized with 10 g/L MgCl_2_, and then abalones were randomly divided into challenged and negative control groups of 180 and 70 individuals, respectively. For the challenged group, 100 μL of viral inoculum (adjusted at 10^4^ copies of viral DNA/μL) was injected into the pedal muscle of 180 abalones: 150 of them were maintained in three 50 L tanks and used for serial sampling whereas 30 of them were placed in three 18 L tanks and used to record survival. For the negative control group, 100 μL of control homogenate was injected. A total of 40 animals were maintained in one 50 L tanks and used for serial sampling, whereas 30 were placed in three 18 L tanks and used to record survival.

### Animal sampling and HaHV-1 DNA quantification

Six (2 abalones per tank) and 3 abalones were sampled at 0, 12, 24, 30, 36, 48, 60 and 72 hours post injection (hpi) from challenged and negative control groups, respectively. Four types of tissue (mantle, gill, hepatopancreas and neural tissue surrounded by some muscle) were dissected from each individual and divided in 2 pieces for DNA and RNA extraction. DNA extraction was performed from tissues and homogenates with a TIANamp^TM^ Marine Animals DNA Kit (Tiangen Biotech, China), according to the manufacturer’s protocol. The purity and quantity of the isolated DNA was determined with a Nanodrop 2000 spectrophotometer (Thermo Fischer Scientific, Germany).

HaHV-1 DNA quantification was carried out by qPCR targeting ORF66 (annotated as unknown protein) and using a protocol adapted from the World Organization for Animal Health (OIE) Manual of Diagnostic Tests for Aquatic Animals, 2017. Briefly, amplification was performed in 25 µl reactions containing 12.5 µl of 2x FastStart Essential DNA Probes Master (Roche Diagnostics, Swiss), 1 µl of each primer (10 µM), 0.5 µl of TaqMan® probes targeting the viral ORF66 (10 µM), 2 µl of template DNA and 8 µl of water. The PCR assay was performed using a Bio-Rad CFX Connect Real-Time system (Bio-Rad Laboratories, USA) and run under the following conditions: 1 cycle 95 °C for 10 min, followed by 40 cycles of amplification at 95 °C for 10 s, 60 °C for 20 s. The virus quantitation was carried out by comparison with a standard curve, which was created from a 10-fold dilution series (10^8^−10^1^ copies µl^−1^) of plasmid containing the target sequence. A qPCR negative control was carried out with 2 µl of deionized water instead of DNA sample. Each sample was tested in duplicate and it was recorded as positive if both replicates were positively amplified. We estimated the HaHV-1 infection burden of each sample as the mean genomic equivalent (GE) score (ng^−1^ of total DNA) of the duplicates.

### RNA extraction and sequencing

Due to the excessive polysaccharide content of abalone samples, attempts to extract high quality RNA for high-throughput sequencing failed in our laboratory. Therefore, 3 mantle samples at 60 hpi were sent to Beijing Novogene Technology Co. Ltd. (China) for RNA extraction and whole transcriptome sequencing based on Illumina technology. A total amount of 1.5 µg RNA per sample was used as input material for the RNA sample preparations. Sequencing libraries were generated using NEBNext^®^ Ultra™ RNA Library Prep Kit for Illumina^®^ (NEB, USA) following the manufacturer’s recommendations and index codes were added to attribute the reads to each sample. Briefly, mRNA was purified from total RNA using poly-T oligo-attached magnetic beads (NEB). Fragmentation was carried out using divalent cations under elevated temperature in NEB NextFirst^®^ Strand Synthesis Reaction Buffer (5x). First strand cDNA was synthesized using random hexamer primers and M-MuLV Reverse Transcriptase (RNase H^−^) (NEB). Second strand cDNA synthesis was subsequently performed using DNA Polymerase I and RNase H (NEB). Remaining overhangs were converted into blunt ends via exonuclease/polymerase activities (NEB). After adenylation of the 3′ ends of DNA fragments, NEBNext^®^ Adaptor with hairpin loop structure were ligated to prepare for hybridization. To select cDNA fragments of preferentially 250~300 bp in length, the library fragments were purified with AMPure XP system (Beckman Coulter, USA). Then 3 µl USER Enzyme (NEB, USA) was used with size-selected, adaptor-ligated cDNA at 37 °C for 15 min followed by 5 min at 95 °C before PCR. Subsequently, PCR was performed with Phusion High-Fidelity DNA polymerase (NEB), Universal PCR primers and Index (X) Primer (NEB). At last, PCR products were purified (AMPure XP system) and library quality was assessed on the Agilent Bioanalyzer 2100 system. The clustering of the index-coded samples was performed on a cBot Cluster Generation System using TruSeq PE Cluster Kit v3-cBot-HS (Illumina) according to the manufacturer’s instructions and sequencing was carried out on an Illumina Hiseq platform (2 × 150 paired-end reads).

### Viral genome sequencing

To reduce the noise of host DNAs, a Long-Range PCR (LR-PCR) based approach was used to enrich HaHV-1 DNA for Pacbio sequencing. Twenty one primer pairs were designed with the online version of GenoFrag based on the genome sequence of HaHV-1 Taiwan variant (GenBank accession no. KU096999)^43^. The length of LR-PCR products was set at 9–15 kb and overlapped the adjacent ones by 500–1500 bp. DNA was extracted from challenged samples using the Qiagen DNeasy Blood and Tissue kit (Qiagen, USA) according to the manufacturer’s protocol. PCR was performed in a 50 μl reaction volume that included 10 µL 5x PrimeSTAR GXL Buffer, 4 µL dNTP Mixture (2.5 mM each), 1 µL PrimeSTAR GXL DNA Polymerase (Takara, Japan), 1 µL each of forward and reverse primers (10 µM, listed in Supplementary Table 6), 2 µL DNA template and 31 µL of PCR-grade H_2_O. LR-PCR was performed using Veriti Thermal Cycler (Applied Biosystems) under the following conditions: 94 °C for 1 minutes, followed by 35 cycles of amplification (denaturation 98 °C, 10 s; annealing 50 °C, 15 s, extension 68 °C, 10 minutes) and hold at 4 °C. PCR product sizes were detected on 0.8% 1 × TAE agarose gels stained with GeneFinder™ (Zeesan Biotechnology Inc.). PCR amplicons were purified with QIAquick PCR Purification Kit (Qiagen) and quantified using Qubit Fluorometer (Life Technologies). Then, the amplicons were mixed in equimolecular proportions, and 10 µg of the mixtures were send for genome sequencing and assembly at Guangzhou Gene Denovo Biotechnology Co., Ltd. Sequencing was performed on the PacBio RS II sequencer with 10 kb SMRTBell library prepared with manufacturer’s specification (Pacific Biosciences, USA).

### Bioinformatic analyses

If not differently indicated, all the analyses were performed using CLC Genomic Workbench v.11.0 (Qiagen). Raw reads were trimmed for the presence of adaptor sequences and for quality using TrimGalore! (https://www.bioinformatics.babraham.ac.uk/projects/trim_galore/), allowing a maximum of 2 ambiguous bases and a quality threshold of Q20. The 8 available *Malacoherpesviridae* genomes were retrieved from the NCBI database (IDs: AY509253, GQ153938, KY242785, KY271630, MG561751, KP412538, NC018874 and KU096999). High quality (HQ) reads were mapped on all *Malacoherpesviridae* genomes (with similarity and length mapping parameters ranging between 0.8/0.5 and 0.9/0.9, respectively) and mapped reads were labeled as ‘*Malacoherpesviridae* reads’ and retained for subsequent analyses. A *large gap read mapping* tool was employed to identify spliced reads, i.e. reads mapping on the reference genome with an internal gap.

### Analysis of DNA reads

Firstly, the resulting Pacbio reads longer than 500 bp with a quality value over 0.75 were merged together into a single dataset. Then, the hierarchical genome-assembly process (HGAP) pipeline^44^ was used to correct for random errors in the long seed reads with a threshold of 6 Kb, by aligning shorter reads against them. Finally, *de novo* assembly was performed using the corrected and preassembled reads by Celera Assembler with an overlap-layout-consensus (OLC) strategy^45^. Since SMRT sequencing introduce very little variations of the quality throughout the reads, no quality values were used during the assembly. To validate the quality of the assembly and determine the final genome sequence, the Quiver consensus algorithm was used^44^.

### De-novo and viral protein characterization

*De-novo* assembly of ‘*Malacoherpesviridae* reads’ was performed with the CLC assembler tool, setting word and bubble size parameters to ‘automatic’ and a minimal contig length of 500 bp; the assembled contigs were subjected to open reading frame (ORF) prediction with the NCBI ORF finder tool (https://www.ncbi.nlm.nih.gov/orffinder/), setting a minimal length of 100 codons according to the criteria described by^2^ and used for the annotation of all *Malacoherpesviridae* genomes. Briefly, overlapping ORFs and ORFs shorter than the minimal length were retained if displayed features supporting their effective existence, like conserved domain(s), a transmembrane or a signal peptide region. SignalP and HMMer^46^ were used to identify a signal peptide region or the presence of conserved protein domains, respectively (using the Pfam-A models, v.29^47^, with a cut-off *E-value* of 0.01).

### Identification of variable positions on viral genome

Single Nucleotide Polymorphism (SNP) analysis was separately performed on the 3 mapping files (produced mapping the reads on KU096999.1 genome reference). Nucleotide changes were called ‘SNP’ if present at least in 5% of the locally aligned reads using the following parameters: minimum average quality of the five surrounding bases, 30; minimum quality of central base, 30; minimum required coverage, 50x. InDels were identified using an algorithm that first identifies positions in the mapping files with an excess of reads with unaligned ends. Once these positions and the consensus sequences of the unaligned ends were determined, the algorithm maps the consensus sequences to the reference sequence around other positions with unaligned ends. Subsequently, the consensus assembled from the DNA reads were used to validate SNPs and InDels, by mapping them on the reference genome and manually inspecting the positions of all the predicted variations.

### *In-silico* expression analysis

To quantify the expression of viral ORFs, HQ RNA reads were mapped on the improved version of the HaHV-1 genome (KU096999.1), setting both length and similarity parameters to 0.9. Starting from the read counts per ORF, Transcripts Per Million (TPM) expression values were computed according to^48^.

### Viral gene expression analysis

To further study the viral gene expression over the time course experiment, 37 primer pairs for qRT-PCR analysis were designed using Primer-BLAST (https://www.ncbi.nlm.nih.gov/tools/primer-blast/) (Supplementary Table 7). These 37 ORFs were selected according to their expression levels of the RNA-seq data at 60 hpi. The efficiency of each primer pair was verified to be within the range of 90–110% of efficiency by constructing a standard curve from serial dilutions (Supplementary Table 7).

For qRT-PCR, total RNA was prepared in our lab using TRIzol reagents (Invitrogen, USA) in accordance with the manufacturer’s instructions. cDNA was synthesized from 2 μg of total RNA with reverse transcriptase (Takara, Japan) and random primers. qRT-PCR was performed in a total of 20 μL reaction system using TB Green™ Premix Ex Taq™ II (Takara, Japan) based on CFX Connect™ Real-Time System (Bio-Rad Laboratories, Inc.). Each reaction system contained 10 μL of TB Green Premix Ex Taq (Tli RNaseH Plus), 0.4 ul of ROX Reference Dye II, 0.8 ul of each primer at the final concentration of 400 nM each, 6 ul of distilled water, and 2 ul of cDNA dilution (1/20). qPCR reactions were performed under the following thermal cycling conditions: 1 cycle of 95 °C for 30 s, followed by 40 cycles of 95 °C for 5 s, 60 °C for 30 s and a melt curve step (from 65 °C, gradually increasing 0.5 °C/s to 95 °C, with acquisition data every 1 s). The relative expression levels of viral genes among samples were normalized to the abalone cytochrome c oxidase subunit I (COX I), which has been verified as reliable internal standard (unpublished data). All reactions were performed in triplicates and the data were calculated as the mean of relative mRNA expression using the 1/delta Ct method^49^ and were therefore reported in a scale for 0 to 1.

### Phylogenetic analysis

Phylogenetic analysis was carried out on 40 concatenated homologue ORFs retrieved from the 3 HaHV-1 genomes. Briefly, the homologue ORFs were retrieved from the Australian viral variant (NC018874), from the improved version of the Taiwanese variant herein described (KU096999.1) and from the genomic consensus obtained through PacBio sequencing. ORFs were concatenated and aligned using MUSCLE^50^ and a phylogenetic tree was produced based on NJ algorithm and Jukes-Cantor distance measurement, applying 1,000 bootstrap replicates.

### Accession codes

The high-quality RNA-Seq reads and raw PacBio reads are available through the SRA database under accession numbers PRJNA47124 and PRJNA492770 respectively.

## Electronic supplementary material

Supplemental infomation

Supplementary Dataset 1

Supplementary Dataset 2

Supplementary Dataset 3

Supplementary Dataset 4

Supplementary Dataset 5

Supplementary Dataset 6
