# Supplementary material for: RNA-seq of HaHV-1-infected abalones reveals a common transcriptional signature of Malacoherpesviruses
Source: Sci Rep. 2019 Jan 30;9:938. doi: 10.1038/s41598-018-36433-w (PMC6353905; doi:10.1038/s41598-018-36433-w)
Supplement: Supplementary file 1 — Supplemental infomation [file 41598_2018_36433_MOESM1_ESM.docx]

**Supplemental Materials**

**Title
RNA-seq of HaHV-1-infected abalones reveals a common transcriptional signature of *Malacoherpesviruses***

**Authors**Chang-Ming Bai^1,+^, Umberto Rosani^2,+^, Ya-Nan Li^1,3^, Shu-Min Zhang^1,4^, Lu-Sheng Xin^1^, Chong-Ming Wang^1,^*

**Affiliations**

^1^ Key Laboratory of Maricultural Organism Disease Control, Ministry of Agriculture; Laboratory for Marine Fisheries Science and Food Production Processes, Qingdao National Laboratory for Marine Science and Technology; Qingdao Key Laboratory of Mariculture Epidemiology and Biosecurity; Yellow Sea Fisheries Research Institute, Chinese Academy of Fishery Sciences, Qingdao 266071, China

^2^ Department of Biology, University of Padua, Padua 35121, Italy

^3^ College of Fisheries, Tianjin Agriculture University, Tianjin 300380, China

^4^ College of Fisheries and Life Science, Dalian Ocean University, Dalian 116023, China

**Contents**

Supplementary Figure 1

Supplementary Table 1

Supplementary Table 2

Supplementary Figure 2

Supplementary Figure 3

Supplementary Figure 4

Supplementary Table 6

**
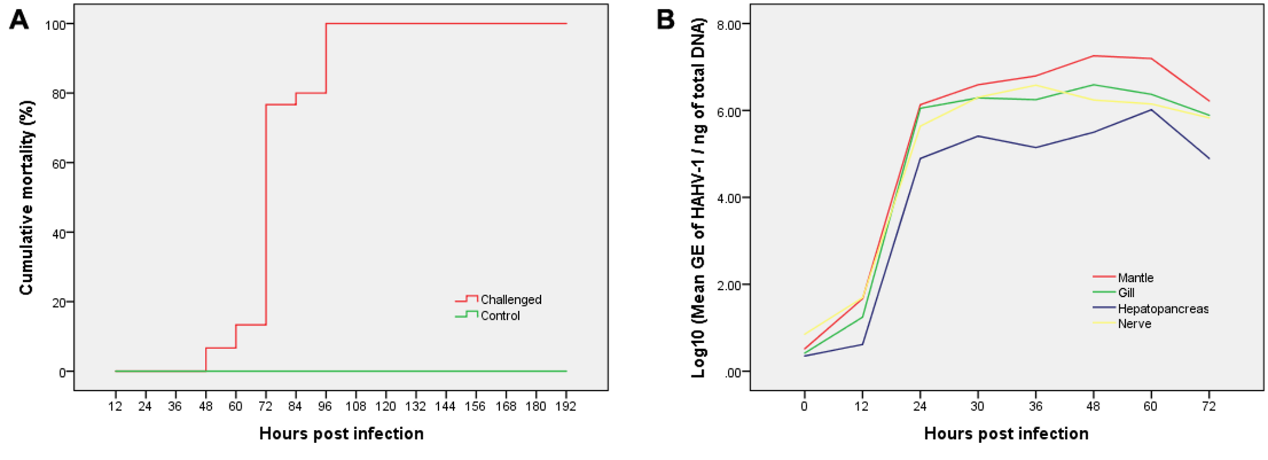
**

**Supplementary Figure 1.** A. Cumulative mortality curve over the infection period (reported in hours) for challenged and control abalones. B. Viral DNA over the infection period. Log_10_ of genomic equivalents (GE) of HaHV-1 per ng of total DNA measured in mantle, gill, hepatopancreas and nerve were reported.

**Supplementary Table 1.** Mapping of HQ reads on the 8 *Malacoherpevirus* genomes. NCBI ID, description and consensus length of each genome were reported as well as the number of total, single and paired mapped reads. Mapping length and similarity parameters were both set at 0.9.

| **ID** | **Description** | **Consensus length** | **Mapped reads** | | |
| --- | --- | --- | --- | --- | --- |
|  |  |  | **Total** | **Single** | **In pairs** |
| AY509253 | OsHV-1 | 207439 | 0 | 0 | 0 |
| GQ153938 | ANVN | 210993 | 0 | 0 | 0 |
| KY242785 | OsHV-1-microvariant | 204886 | 0 | 0 | 0 |
| KY271630 | OsHV-1-microvariant | 204897 | 0 | 0 | 0 |
| MG561751 | OsHV-1-microvariant | 203983 | 0 | 0 | 0 |
| KP412538 | OsHV-1-Scapharca | 199354 | 0 | 0 | 0 |
| NC018874 | HaHV-1-AUS | 211518 | 3,282,113 | 360,231 | 2,921,882 |
| KU096999 | HaHV-1-TAI | 199102 | 9,778,838 | 665,214 | 9,113,624 |
|  |  | **Totals** | **13,060,951** | **1,025,445** | **12,035,506** |

**Supplementary Table 2.** Mapping of HQ reads on HaHV-TAI genome (KU096999.1). Different mapping algorithms and parameters were applied.

| **Datasets** | **Algorithm** | **Parameters**  **(length and similarity)** | **No . of mapped reads** |
| --- | --- | --- | --- |
| MA49+ MA50+  MA51 | Simple mapping | 0.9 / 0.9 | 12.747 M |
|  | Simple mapping | 0.5 / 0.8 | 13.085 M |
|  | Large gap read mapping | 0.9 / 0.9 | 12.379 M |

**
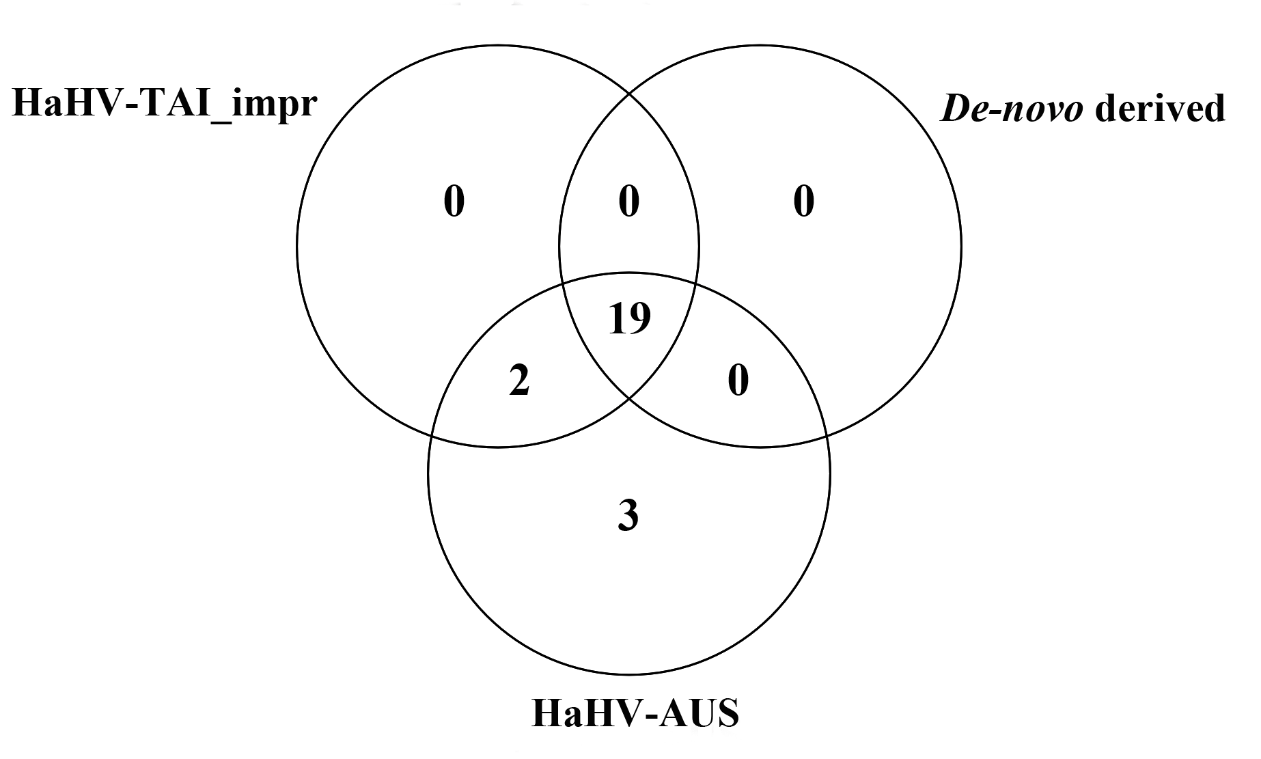
**

**Supplementary Figure 2.** The Venn diagram depicted the number of conserved domains found on HaHV-TAI_impr, on de-novo-derived ORFs and on HaHV-AUS

**
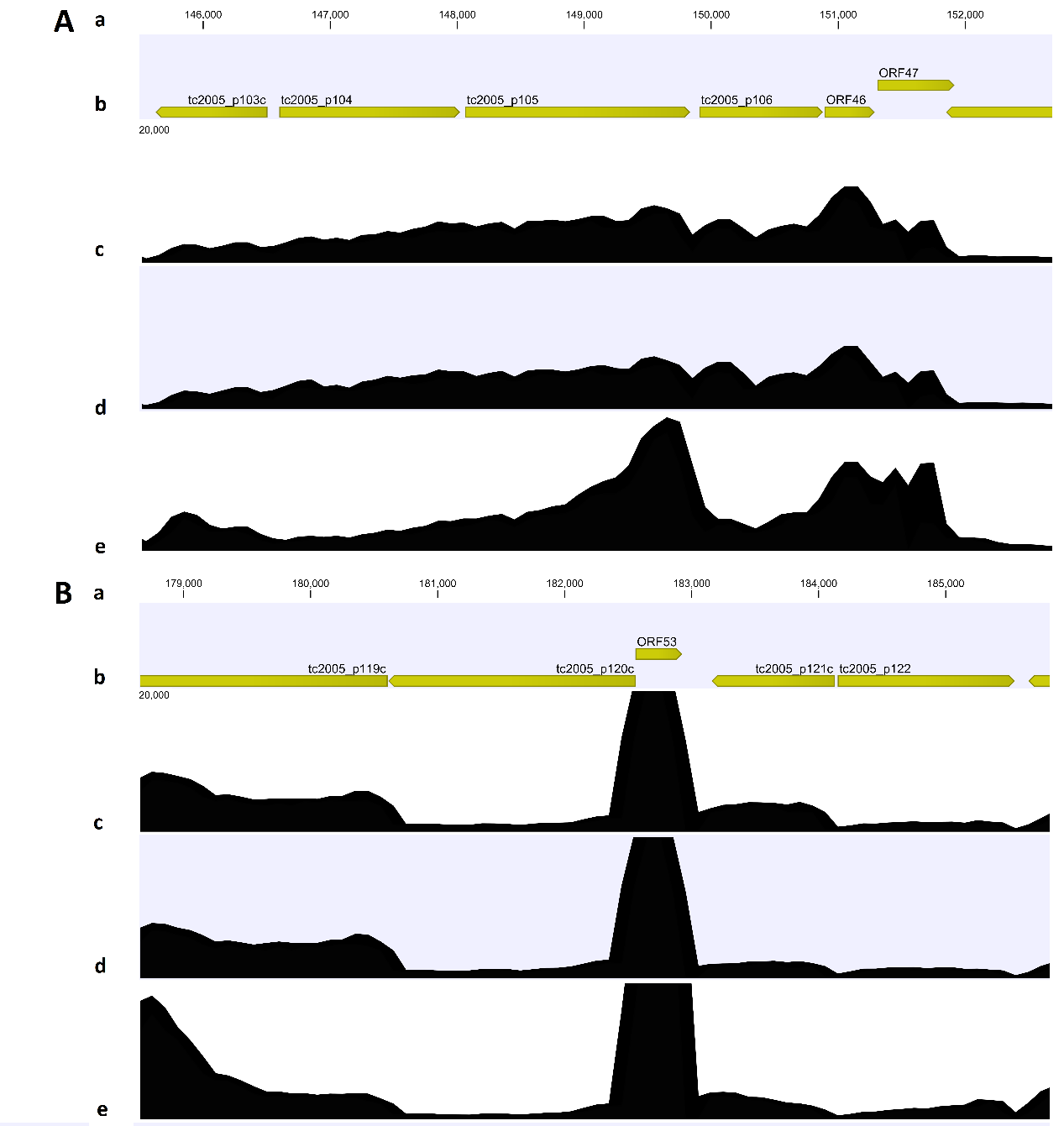
**

**Supplementary Figure 3. A.**Coverage graphs for ORFs p103-p104-p105-p106 in the 3 RNA-seq samples. The even distribution of coverage along the 4 predicted ORFs suggested their co-transcription in a polycistronic mRNA. **B.** Coverage graphs of ORF53 (unknown) taken as example of a viral ORF with well-defined transcription boundaries.

a. HaHV-1 genome; b. predicted ORFs; c, d and e coverage graphs for sample MA49, MA50 and MA51.


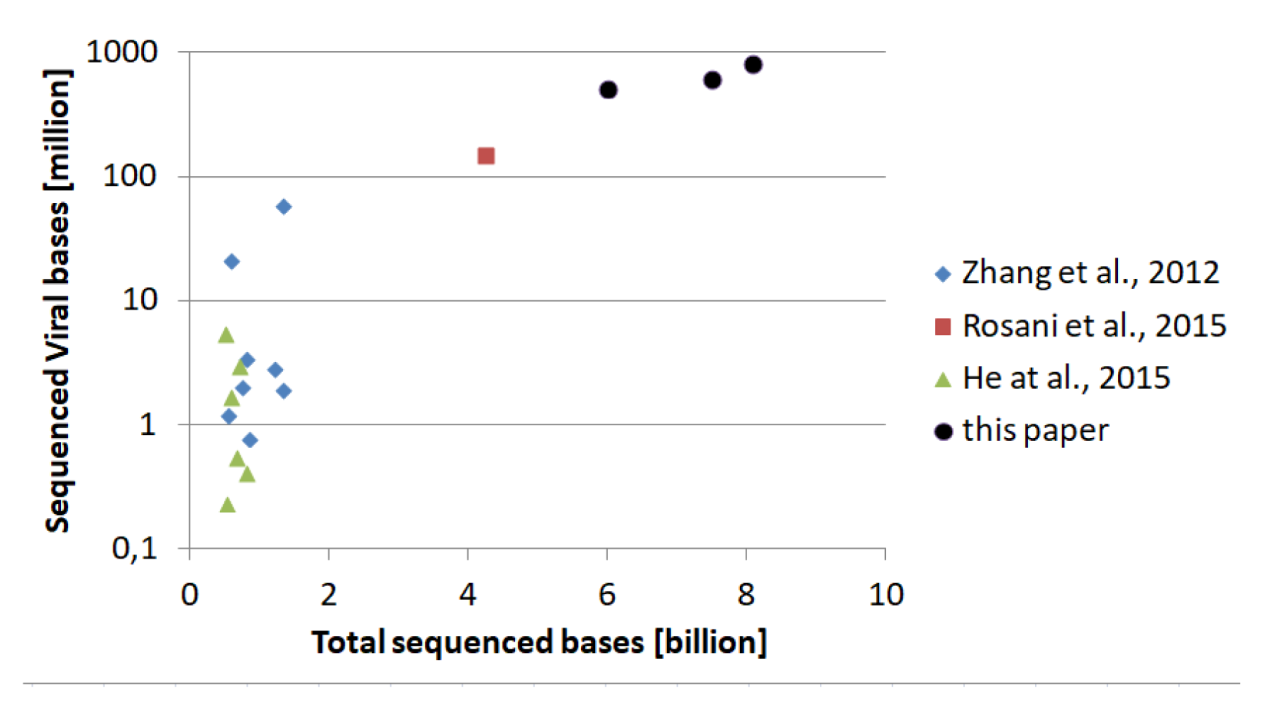


**Supplementary Figure 4**. Distribution of total sequenced bases (in billions) versus viral sequenced bases (in millions) for dual RNA-seq experiments involving Malacoherpesviruses ^1–3^.

**Supplementary Table 6.** Primer sequences and primary characteristics for long-range PCR

| Primer ID | Primer sequences | Start position | End  position | Amplicon  size (bp) |
| --- | --- | --- | --- | --- |
| AbGF0-1F | GGAGGAGAGAGGATGTGTAAGGTAA | 977 | 1021 | 13,910 |
| AbGF1R | GCACAAACCCAACCTCTATCTCTTC | 14882 | 14906 |  |
| AbGF2F | GCCAAGTCGTAGTTTCTCAGTTCTC | 14393 | 14417 | 10,731 |
| AbGF2R | GCAGTTGTAGAAAGCGTGAAGTCGT | 25099 | 25123 |  |
| AbGF3-1F | GCCCGCTTCCATATCTTCCTCTAAA | 23606 | 23630 | 10,937 |
| AbGF3-3R | CTTCCTGCTGCTCTGCTTGACTTAT | 34542 | 34566 |  |
| AbGF4-2F | GGTCGTTGGTTAGTGGAGTAGATGA | 32957 | 32981 | 10,158 |
| AbGF4-1R | CCCTGTTTCTGATGAGAGTGTGAGT | 43114 | 43138 |  |
| AbGF5F | CATCGGTACTACTCATATCCTCCTC | 42786 | 42810 | 10692 |
| AbGF5R | GCGGAGACGAAGTTTGAGTTACGAT | 53453 | 53477 |  |
| AbGF6-1F | CCAGAAGGACGGAGAAGTTGAAGAT | 53062 | 53086 | 9,349 |
| AbGF6-1R | CCGTCATCCCGCTTAATCAAGTCTT | 62386 | 62410 |  |
| AbGF7-1F | CCGCTACTGGATTTGTTGTTGGTGA | 60884 | 60908 | 10796 |
| AbGF7-1R | CGTTTGTCTGACCTACCCTACCTTA | 71655 | 71679 |  |
| AbGF8-1F | GGCGTGGGAAGTAAAGGTAAATCTC | 71020 | 71044 | 11,603 |
| AbGF8-2R | CGATCTGCTCTTCTACTCTCTTCAC | 82598 | 82622 |  |
| AbGF9-2F | CCGCCATCACTCAACTATCTACATC | 81993 | 82017 | 10,966 |
| AbGF9-2R | GTGCTGTACCTGGAAACCTTTGTGT | 92934 | 92958 |  |
| AbGF10-2F | TCCCGTGTTGAATTTGCTGGCGTAA | 91897 | 91921 | 9,887 |
| AbGF10-1R | GCGACGGAACCATTGAGATTTGTGA | 101759 | 101783 |  |
| AbGF11-2F | CACCCTGTTAATCTGCCTGTCTATC | 100972 | 100996 | 10,924 |
| AbGF11-1R | GATCGTCAGCAGTACAAGGTCTTCA | 111871 | 111895 |  |
| AbGF12-2F | TGGGACCTACTAGCTTGTTCATCTC | 111556 | 111580 | 10,559 |
| AbGF12-2R | GCCTTCTCAATCCGTTCCAGTATAC | 122090 | 122114 |  |
| AbGF13F | AGGCGGGTCCCAAACACACAATAAT | 120204 | 120228 | 10,834 |
| AbGF13R | TCCTTTCTCCACTTCCTTGACTCAG | 131013 | 131037 |  |
| AbGF14F | GCGAGGAAACTCAAGAGAACACAAC | 130236 | 130260 | 10,687 |
| AbGF14R | CTTCCTTCACTCCCTCTCTCAAACA | 140898 | 140922 |  |
| AbGF15-1F | CCATCCTAACCTTAATCGGCATCTG | 140503 | 140527 | 11,257 |
| AbGF15-2R | CGAGGAAGCAGAAAGTTTGAGTCAG | 151759 | 151783 |  |
| AbGF16-2F | CCCGCCAAAGTGATAGAGGTTAAAG | 148543 | 148567 | 11,622 |
| AbGF16-1R | ACGCCTATGCTGGTGAGCTTTCTAT | 160164 | 160188 |  |
| AbGF17-2F | AACGAGACTGACGCTGAACTACACA | 159496 | 159520 | 11,439 |
| AbGF17-2R | ATGCTTCTTCACGCCGTTCGATACT | 170910 | 170934 |  |
| AbGF18F | CGATCCGCTGGAAGACAAAGAGTTT | 169314 | 169338 | 10,816 |
| AbGF18R | TCCCTCGTCTCCGAAATGAATCTTG | 180105 | 180129 |  |
| AbGF19-2F | GAGGGTTCTCATGGTACGATTCTTG | 178837 | 178861 | 10,710 |
| AbGF19-2R | GCCTTGCGTGTTATCATTCGTACTG | 189522 | 189546 |  |
| AbGF20-1F | CTTCCCTGACCTCGTTGATTACACA | 188059 | 188083 | 10,894 |
| AbGF20-1R | TCCTGCGTGTCTTCATCTTGAGTCA | 198928 | 198952 |  |
| AbGF21-2F | GCAAATCACCCAGTATGGCAATCCT | 198103 | 198127 | 13,389 |
| AbGF22-1R | AAGGCGGGTCCCAAACACACAATAA | 211467 | 211491 |  |

**References**

1. He, Y. *et al.* Transcriptome analysis reveals strong and complex antiviral response in a mollusc. *Fish Shellfish Immunol.* **46,** 131–144 (2015).

2. Rosani, U. *et al.* Dual analysis of host and pathogen transcriptomes in ostreid herpesvirus 1-positive *Crassostrea gigas*. *Environ. Microbiol.* **17,** 4200–4212 (2015).

3. Zhang, G. *et al.* The oyster genome reveals stress adaptation and complexity of shell formation. *Nature* **490,** 49–54 (2012).
